# The Dry Treatment of Chronic Suppurative Inflammation of the Middle Ear

**Published:** 1884-12

**Authors:** 


THE DRY TREATMENT OF CHRONIC SUPPURATIVE INFLAMMATION OF THE MIDDLE EAR. By Charles J. Sundy, A. M., M. D.—This pamplet describes a very unique mode of treatment for tympanic inflammation, and a number of cases are refered to as examples of the beneficial effects of such treatment.

